# Bioavailability of zinc from different organic zinc chelates and their effect on the growth, whole body, tissue zinc content, enzymes activity and proximate composition of *L. rohita*

**DOI:** 10.1371/journal.pone.0314146

**Published:** 2025-03-19

**Authors:** Muhammad Tahir, Noor Khan, Mahroze Fatima, Naveed Ul Haque, Simon J. Davies

**Affiliations:** 1 Department of Fisheries and Aquaculture, University of Veterinary and Animal Sciences, Lahore, Pakistan; 2 Department of Animal Nutrition, University of Veterinary and Animal Sciences, Lahore, Pakistan; 3 Aquaculture and Nutrition Research Unit (ANRU) Carna Research Station, Ryan Institute, School of Natural Sciences, University of Galway Carna Co., Galway, Ireland; Rani Lakshmi Bai Central Agricultural University, INDIA

## Abstract

Minerals play an essential role in aquatic animals to maintain their normal physiological and metabolic functions. This feeding trial assessed the impact of various zinc sources on *L. rohita* performance. Dietary treatments included a Control group with zinc sulfate (226.25 mg/kg), and treatment groups with zinc citrate (150.15 mg/kg), zinc acetate (230.56 mg/kg), zinc monomethionine (244.75 mg/kg), and zinc gluconate (395.94 mg/kg). In 15 aquaria, 300 fish (15.84 ± 0.07g) were randomly distributed in triplicate groups. Results revealed significantly (P < 0.05) higher final body weight (FBW), feed conversion ratio (FCR), body weight gain (BWG), and sediment growth rate (SGR) for zinc gluconate fed fish compared to the other sources. Zinc gluconate fed *L. rohita* also exhibited the lowest feed intake, while initial body weight (IBW) and survival rate (SR %) did not significantly differ. Proximate fish analysis showed non-significant (P > 0.05) differences among treatments. Tissue zinc analysis demonstrated significantly (P < 0.05) higher zinc content in *L. rohita* receiving zinc gluconate. Antioxidant enzyme activity indicated lower (2.12 ± 0.01) thiobarbituric acid reactive substances (TBARS) in the zinc gluconate treatment, with elevated levels of key biomarker enzymes, glutathione peroxidase (GPx), catalase (CAT), and superoxide dismutase (SOD). Stress and immune response indicators revealed significantly lower hematocrit (HCT), cortisol (CRT), and glucose (GLU) levels in zinc gluconate fed fish, while CRT was higher (36.62 ± 0.65 mg/ml). Blood serum parameters, including alanine aminotransferase (ALT), aspartate aminotransferase (AST), and alkaline phosphatase (ALP), remained lower (29.65 ± 0.85 U/ml, 84.76 ± 2.45 U/ml, and 212.87 ± 6.14 U/ml), in fish fed zinc gluconate respectively. In conclusion, different organic zinc sources, particularly zinc gluconate, improved *L. rohita* growth, tissue zinc concentration, antioxidant enzyme activity, blood serum parameters, and stress and immune response to varying extents.

## Introduction

Dietary mineral content is crucial for maintaining normal physiological and metabolic functions in all aquatic animals. This topic has also been reviewed for fish [1], detailing species-specific requirements informed by a decade of advancements. A feed with a balanced nutrient composition is essential for ensuring fish quality and the commercial success of intensive aquaculture production at the profitable cost. A major issue faced by the intensive culture systems is eutrophication caused by using excessive minerals in fish feed [2]. To resolve this issue, mineral-balanced diets are required according to the requirements of the species to avoid leaching excess mineral contents in the aquatic environment. Zinc (Zn) is an essential micro-mineral needed for the proper growth, development, metabolic activity, and immunological function of fish [3]. Zinc is an essential cofactor for multiple antioxidant enzymes, including superoxide dismutase, This enzyme complex plays a crucial role in antioxidant defense by neutralizing harmful free radicals, thereby protecting tissues and organs from damage and supporting immune function [4,5]. As such, Zn stimulates growth and is involved in various cellular activities, including cell proliferation, immune function, cofactor replication, and free radical production. [6] Highlighted the synergistic effects of various dietary Zinc forms used as supplements, and their interaction with dietary protein levels, on the growth performance, intestinal integrity, immune function, and oxidative stress response in Nile tilapia (*Oreochromis niloticus*). This interaction reveals a complex interplay where the optimal combination of Zinc and protein significantly enhances the species’ overall health, resilience, and vitality by maximizing nutrient absorption and biological efficiency. On the other hand, it’s catalytic, structural, and regulatory functions affect the synthesis of nucleic acid [7,8] and protein metabolism [9]. Although Fish acquire zinc primarily through osmoregulation, a process involving the constant exchange of water and ions between the fish’s body and its aquatic environment. Specialized cells in the gills, the primary respiratory organs, actively transport zinc ions from the surrounding water into the bloodstream. This uptake is influenced by factors such as water quality, zinc concentration, and the specific physiological requirements of the fish species, insufficient Zn concentration in the aquatic environment leads to inadequate fish growth [10] low survival rate, cataracts [11] dwarfism, bone weakness and skeletal deformities [12,13]. Excessive zinc levels regulate toxicity, and impaired reproductive functions [14]. However, it is apparent that optimal Zn level stimulates growth performance and improved the overall health of fish as reported by several authors in the literature [15,16].

Zn sulphate (ZnSO_4_.7H_2_O) is the inorganic mineral source used more commonly to fulfill the dietary Zn requirements of fish. However, a more stable structure and low molecular weight of organic minerals prevent mineral binding and increase their uptake and assimilation in the fish intestine [17]. Organic mineral utilization is a more natural process of micro mineral utilization as feed ingredients will contain minerals comprised of mineralized protein and amino acids within the protein matrix [18].Interestingly, seawater [19] and freshwater [20] are sub-optimal with the necessary Zn concentration to attain a desired Zn intake for fish. Consequently, a balanced dietary zinc formulated diet is more favorable to fulfill the zinc requirement in intensive aquaculture conditions. Fish absorb and utilize Zn directly from the environment or from the feed. Each fish species has its own specific Zn requirement; Nile tilapia [21] grouper [22] channel catfish [23] revealed a 15 to 40 mg/kg zinc requirement in their respective investigations. However, dietary Zn requirement of juvenile grouper [24], rainbow trout [25], varied from 28.9-33.7 mg/kg and 80 mg/kg diet respectively.

Furthermore, metal-amino acid complexes could have superior efficacy as a replacement of inorganic trace minerals in commercial diets for Nile tilapia [26]. However, dietary organic Zn requirement has not been determined until now for *L. rohita* juveniles. Therefore, the purpose of the present study was to evaluate the bioavailability of Zinc from different organic Zn chelates (citrate, acetate, monomethionine, gluconate) as compared to the inorganic Zn (sulphate) and their effect on growth performance, whole body proximate composition, different body organs, enzymes activity, Immune assay and Blood biochemical parameters of *L. rohita*.

Major carps (*L. rohita, Catla catla, and Cirrhinus mrigala*) shows a huge contribution in freshwater production of the subcontinent. Unique flavor, rapid growth and disease resistance in *L. rohita* directly influence market demand by impacting production costs, supply stability, and ultimately, consumer prices [27]. This species holds paramount importance in numerous Asian countries, especially those in the Indian subcontinent. Developing strategies to cultivate robust, disease-resistant fish capable of withstanding stress is paramount for aquaculture sustainability. To support this goal and address economic challenges, it is essential to re-evaluate zinc requirements for *L. rohita* by considering novel trace element products in aquafeed formulations that prioritize fish welfare and environmental responsibility.

## Materials and methods

The experimental study on fingerlings of *L. rohita* was conducted in the nursery fish rearing unit, Department of Fisheries & Aquaculture, University of Veterinary and Animal Sciences, Ravi Campus, Pattoki, Pakistan. This study was approved by the Institutional Animal Care and Use Committee and adhered to the university’s ethical guidelines. All protocols were in accordance with international standards for fish experimentation.

### Experimental design and feed formulation

In the study, inorganic Zn sulphate and four different organic Zn chelates were added to a basal diet and all diets were fed to the fish for a 90-day period for performance assessment. A total of 5 dietary treatments were made in such a way that Zinc sulphate was assigned as the control group containing the basal diet with the addition of Zn sulphate at an inclusion of 226.25 mg/kg. Furthermore, in other experimental treatments, the basal diet was supplemented separately with zinc citrate, zinc acetate, zinc methionine, and zinc gluconate at inclusion levels of 150.15, 230.56, 244.75, and 395.94 mg/kg, respectively. These levels were calculated to provide a target available zinc content of 51.42 mg/kg, considering the molecular weight and zinc concentration of each zinc source (Table 1). Furthermore, the optimal dietary requirement of inorganic zinc sulfate for *L. rohita* is 51.42 mg/kg [28].

**Table 1:** Experimental feed formulation and ingredients composition.

Ingredients	Zinc sulphate	Zinc citrate	Zinc acetate	Zinc monomethionine	Zinc gluconate
Corn (g/kg)	260	260	260	260	260
Fish Meal (g/kg)	80	80	80	80	80
Soybean Meal (g/kg)	190	190	190	190	190
Wheat Bran (g/kg)	120	120	120	120	120
Rice Polish (g/kg)	180	180	180	180	180
Corn Gluten 60% (g/kg)	130	130	130	130	130
Vitamin& Mineral Premix (g/kg)^*^	10	10	10	10	10
Fish oil (g/kg)	30	30	30	30	30
Zinc (mg/kg)	226.25	150.15	230.56	244.75	395.94
Nutrient Composition					
Dry Matter (%)	90.72	90.78	91.32	90.89	91.19
Crude Protein (%)	27.73	27.79	27.91	27.84	27.77
Ether Extract (%)	7.39	7.21	7.40	7.34	7.13
Ash (%)	8.70	8.44	8.66	8.61	8.64
Energy (Kcal)	2904	2921	2933	2907	2918
Analyzed Zinc Content (mg/kg)	48.34	47.65	48.02	49.1	47.98

^*^Each Kg of vitamin& mineral premix contains Vitamin A 15 M.I.U. Vitamin D3 3 M.I.U. Nicotinic acid 25,000 mg; Vitamin B1 5000 mg Vitamin E 6000 IU Vitamin B2 6000 mg; Vitamin K3 4000 mg Vitamin B6 4000 mg Folic acid 750 mg; Vitamin B12 9000 mg Vitamin C 15,000 mg Calcium pantothenate 10,000 mg. Treatments: Zinc sulphate, Zinc citrate, Zinc acetate, Zinc monomethionine, Zinc gluconate. Pro Foods^TM^ Nutrition. As per the inclusion of zinc from its different sources (Sulphate, Citrate, Acetate, Monomethionine, and Gluconate) ensures that fish fed on 51.42 mg kg^-1^ dietary zinc level. Dietary zinc requirement of fingerling Indian major carp, *L. rohita* is 51.42 mg kg^ − 1^ (Musharraf et al. 2019).

Feed ingredients (Table 1) were procured from the local market, Lahore-Pakistan. Dry feed ingredients were sieved (2 mm) after making finely ground powder in a domestic electric grinder (KEN WOOD Model KM 280). Adequate amounts of water were added to make dough which was further pelletized by using meat mincer (ANEX Model AG3060). Pellets were subsequently sun dried and packed in well-sealed plastic zipper packets and stored in refrigerator at 4˚C.

### Fish husbandry

Before starting the feeding trial, the fish were acclimatized for 2 weeks. At the onset of the feeding trial, fish (*N* = 300) of initial weight 15.84 ± 0.17g were stocked in 15 aquaria (20 fish/100L aquaria), so that three replicates of aquaria were used against each test diet. Fish were provided with test diets up to 3% body weight, 7 days a week for 3 months. The daily ration was divided into two proportions and given twice a day. The tank water was partially cleaned after every two hours of feeding and fresh water was added, excess feed and fecal matter were also removed through manual siphoning respectively. The weight of fish was monitored regularly after every two weeks, and feed rationing was adjusted accordingly to their biomass. Aeration was provided round the clock to maintain dissolved oxygen (DO) level at optimum (5.8-7.3 mg/L), temperature (26-28^o^C), pH(6.5-7.5), salinity (<1.0 ppt), ammonia kit (Hanna HI3824), electrical conductivity and total dissolved solids (TDS) were monitored on daily basis by using digital meters/Multi-meter (Hanna Romania).

### Collection of samples and chemical analysis

At the end of the feeding trial, fish were starved for 24 h, anesthetized by using MS 222 and weighed for growth performance metrics [29]. Three ﬁsh from each tank were randomly selected, labelled properly according to their respective treatment diets, euthanized by an overdose of MS 222 according to a schedule 1 method based on UK and EU protocol and homogenized in a meat mincer. The homogenized samples were used for Zn concentration analysis. Another sample of five fish were collected from each tank randomly, anesthetized using 20mg/L dose of MS-222, afterward livers and other organs/tissues were dissected out for the determination of biological indices, antioxidant enzyme activities, TBARS and Zn concentration. Another six fish were taken out from each tank; Whole blood was taken from the caudal vein (a large blood vessel located near the tail) using a syringe and transferring into properly labeled heparinized vacutainers for determining blood indices. Some blood was also taken in un-heparinized vacutainers and allowed to clot for serum removal and stored at -20°C for subsequent biochemical analysis.

### Growth performance

Growth performance was determined at the initiation of the trial, at two-weekly intervals till the termination of the trial. Increase in body weight, weight gain percentage, FCR and specific growth rate were determined using the following formulas.


$$Weightgain=Finalbodyweight\left(g\right)-Initialbodyweght\left(g\right)$$

$$Weightgain\%=\left(Finalweight-initialweight\right)/\left(Initialweight\right)\times 100$$

$$FCR=(Totaldryfeedintake\left(g\right)/(Wetweightgain\left(g\right)$$

$$SGR=\frac{\left(lnFinalbodyweight-lnInitialbodyweight\right)}{Numberofdays}\times 100$$

### Proximate composition

Feed ingredients, experimental diets, and whole-body proximate composition of fish (total ash, crude fat, crude protein, and dry matter) were determined through standard methods of [30]. The crude protein was determined by using a Kjeldhal apparatus (Kjeltec^TM^ 8100, Foss, China). Crude fat was calculated by using the Soxhlet apparatus (Behro test 901745, Germany), crude ash was determined through muffle furnace (Vulcan D-550) at 660°C while the Dry Matter (DM) content was determined by removing moisture through a hot air oven at 105°C. Zn level in feed ingredients, experimental diets and different fish tissues were determined by using atomic absorption spectroscopy (Hitachi ZA3000 Chiyoda Tokyo Japan) after wet digestion. After performing the entire proximate analysis, the energy content of the feed is measured using the indirect formula: Energy (kcal/kg) =  (2.25 ×  Crude Fat) +  (4.00 ×  Crude Protein) +  (4.00 ×  Nitrogen-Free Extract).

### Tissue zinc analysis

Tissues (whole body, eyes, bones, liver, gills, scales, and muscle) for trace mineral analysis were oven dried to a constant weight, digested in 5 ml of concentrated nitric acid, then diluted to 20 ml with deionized water and analyzed by using a Flame Atomic Absorption Spectrophotometer (iCE3000 series, Thermo-Fisher Scientific, USA) for Zn according to the method of [31].

### Antioxidant enzymes and TBARS assay

The TBARS values, superoxide dismutase (SOD) and catalase (CAT) activities were measured in livers [32,33] respectively. However, glutathione peroxidase (GPx) activity was determined [34]. Protein was measured using an appropriate protein quantification method [81– 82] which allows for determining the protein concentration in the liver samples. The resulting protein concentration was then used to express the enzyme activity in units per milligram of protein (U/mg protein).

### Stress and immune response

Blood samples were collected as described previously and used for the estimation of blood glucose level, hematocrit, serum protein, serum cortisol [35] for the determination of stress and immune response.

### Serum biochemistry

The alanine amino transferase (ALT) and aspartate aminotransferase (AST) activity were determined from the samples of collected serum employing a kit method [36]. The alkaline phosphatase activity (ALP) kit catalyses the magnesium-stimulated base hydrolysis of p-nitro-phenyl phosphate to nitrophenolate, and the response was determined in terms of the increased absorbance at 405nm. The rate of hydrolysis linearly depends on the activity of the enzymes in the serum [37].

### Statistical analysis

The collected data were analyzed using the statistical software SAS (version 9.1). Results of the feeding trial were analyzed statistically by using one-way ANOVA. After the occurrence of significant difference (P < 0.05) and comparison of means, Duncan Multiple Range Test was applied to locate these significances between respective treatments [38].

## Results

### Growth performance

The effect of different zinc treatments on the growth performance of fish are given in Table 2. Overall, fish fed on different dietary zinc sources were found significantly (P < 0.05) high for all the parameters recorded except survival. Among all the treatments, dietary supplementation of zinc in Zinc gluconate improved significantly (P < 0.05) the final body weight (FBW) and FCR, while Zinc citrate, Zinc acetate, and Zinc monomethionine showing medium improvement as compared to the control group. Zinc gluconate depicts the higher BWG as compared to the rest of the treatments. Likewise, significantly lowest feed intake was recorded in Zinc acetate, followed by Zinc citrate and Zinc monomethionine, Zinc gluconate while significantly higher feed intake was observed in Zinc sulphate. Moreover, SGR was found significantly (P < 0.05) higher in Zinc gluconate as compared to Zinc monomethionine and Zinc citrate, although Zinc acetate and Zinc sulphate represents the lowest SGR values.

**Table 2:** Influence of different zinc treatments on the growth performance and feed utilization efficiency of *L. rohita* with S.E.

Parameters	Treatments					P-Value
	Zinc sulphate	Zinc citrate	Zinc acetate	Zinc monomethionine	Zinc gluconate	
IBW (g)	15.84 ± 0.07	15.73 ± 0.15	15.81 ± 0.15	15.63 ± 0.22	15.77 ± 0.28	0.94
FBW (g)	55.26 ± 0.03^c^	60.90 ± 0.48^b^	58.44 ± 2.62^b^	61.07 ± 0.51^b^	68.99 ± 0.16^a^	<0.01
BWG (g)	39.42 ± 0.10^c^	45.17 ± 0.58^b^	42.63 ± 2.68^bc^	45.43 ± 0.66^b^	53.22 ± 0.36^a^	<0.01
BWG (%)	248.77 ± 1.80^c^	287.17 ± 5.94^b^	269.72 ± 18.20^bc^	290.77 ± 7.83^b^	337.57 ± 8.00^a^	<0.01
FI (g)	65.07 ± 0.08^a^	60.89 ± 0.35^c^	59.28 ± 0.12^d^	60.31 ± 0.15^c^	62.63 ± 0.17^b^	<0.01
FCR	1.65 ± 0.01^a^	1.34 ± 0.01^b^	1.40 ± 0.10^b^	1.32 ± 0.02^b^	1.17 ± 0.01^c^	<0.01
SGR	1.77 ± 0.00^c^	1.84 ± 0.01^b^	1.81 ± 0.03b^c^	1.84 ± 0.01^b^	1.91 ± 0.00^a^	<0.01
SR (%)	93.33 ± 1.67	96.67 ± 1.67	95.00 ± 2.89	96.67 ± 1.67	98.33 ± 1.67	0.48

Superscripts differ significantly at P ≤  0.05. IBW, initial body weight; FBW, final body weight; BWG, body weight gain; FI, feed intake; FCR, feed conversion ratio; SGR, specific growth rate; SR, survival rate.

### Whole body proximate composition

Data pertaining to the proximate composition of fish fed different zinc sources in diets are depicted in Table 3. It has been observed that different dietary zinc treatments had no significant (P > 0.05) effect on the fish whole body proximate composition.

**Table 3:** Influence of different zinc treatments on the whole-body proximate composition of *L. rohita* with S.E.

Parameters	Treatments					P-Value
	Zinc sulphate	Zinc citrate	Zinc acetate	Zinc monomethionine	Zinc gluconate	
DM%	24.28 ± 0.27	24.14 ± 0.26	23.91 ± 0.26	24.15 ± 0.26	23.92 ± 0.26	0.82
CP%	17.31 ± 0.19	17.16 ± 0.19	17.05 ± 0.18	17.24 ± 0.19	17.25 ± 0.19	0.88
EE%	4.13 ± 0.10	4.18 ± 0.10	4.28 ± 0.11	4.08 ± 0.10	3.96 ± 0.10	0.33
Ash%	2.01 ± 0.05	1.98 ± 0.05	2.06 ± 0.08	2.04 ± 0.08	2.02 ± 0.08	0.94

Superscripts differ significantly at P ≤  0.05. DM, dry matter; CP, crude protein; EE, ether extract.

### Tissue Zinc analysis

Among all the analyzed organs for tissue zinc analysis, a significant difference (P < 0.05) in values for different dietary zinc treatments were recorded as shown in Table 4. Zinc content in the eyes was observed to be higher in Zinc gluconate, medium concentration was observed in Zinc citrate, Zinc acetate and Zinc monomethionine as compared to Zinc sulphate. Similarly, zinc accumulation in scales was found to be higher in Zinc gluconate followed by Zinc monomethionine, Zinc acetate, Zinc citrate, Zinc sulphate, respectively for the fish groups. Bones and muscles show enhanced zinc content in Zinc gluconate however, Zinc monomethionine showed reduced content as compared to Zinc acetate and Zinc citrate, while lowest concentration was observed in Zinc sulphate (control group). Furthermore, liver gave higher values of zinc in Zinc gluconate, Zinc acetate, Zinc monomethionine as compared to Zinc citrate and Zinc sulphate. The gills represent higher zinc content in the Zinc gluconate and Zinc monomethionine as compared to the rest of the treatments. Whereas results of whole-body zinc content revealed significantly higher (P < 0.05) values in Zinc gluconate and decreased values in the Zinc sulphate, Zinc citrate groups of fish as compared to Zinc monomethionine and Zinc acetate.

**Table 4:** Effect of Zn Chelates on Zn content (µg/g) in eyes, scales, bones, muscles, liver, gills and whole body of *L. rohita* with S.E.

Parameters	Treatments					P-Value
	Zinc Sulphate	Zinc citrate	Zinc acetate	Zinc monomethionine	Zinc gluconate	
Eyes	34.31 ± 0.51^c^	37.3 ± 0.52^b^	38.35 ± 0.57^b^	39.27 ± 0.58^b^	43.54 ± 1.16^a^	<0.01
Scales	63.52 ± 1.41^d^	66.66 ± 0.52 cd	68.75 ± 0.63^bc^	72.26 ± 95^b^	91.10 ± 2.11^a^	<0.01
Bones	128.89 ± 2.63^c^	132.59 ± 0.82^bc^	132.67 ± 1.35^bc^	134.95 ± 0.88^b^	144.86 ± 2.18^a^	<0.01
Muscle	43.82 ± 0.77^d^	48.31 ± 0.61^c^	49.44 ± 0.45^c^	52.38 ± 0.49^b^	62.57 ± 1.41^a^	<0.01
Liver	32.35 ± 0.53^c^	32.69 ± 1.15^c^	36.20 ± 0.67^ab^	34.89 ± 0.76^bc^	38.22 ± 0.58^a^	<0.01
Gills	25.23 ± 0.62^d^	28.95 ± 0.33^c^	31.26 ± 0.39^b^	33.88 ± 0.18^a^	34.76 ± 0.54^a^	<0.01
Whole body	48.71 ± 0.14^d^	49.25 ± 0.60^d^	53.04 ± 0.36^c^	56.42 ± 0.18^b^	65.50 ± 0.02^a^	<0.01

Superscripts differ significantly at P ≤  0.05.

### Antioxidant enzymes activity

The effect of different zinc treatments on the TBARS, GPx, CAT, and SOD contents in liver of *L. rohita* is summarized in Table 5. Results revealed significantly (P < 0.05) elevated TBARS content in Zinc sulphate (control) fish which decreased in Zinc acetate, Zinc monomethionine, Zinc citrate as compared to the Zinc monomethionine and Zinc gluconate. Likewise, fish fed the Zinc gluconate diet showed significantly (P < 0.05) higher GPx level compared to the Zinc acetate, although Zinc monomethionine and Zinc citrate presented intermediate activity, but Zinc sulphate (control) showed lowest activity. On the other hand, higher CAT activity was observed in fish fed Zinc gluconate, intermediate in Zinc citrate and Zinc monomethionine compared to the Zinc acetate and Zinc sulphate. Moreover, Zinc gluconate revealed enhanced activity of SOD as compared to the Zinc citrate, Zinc monomethionine, Zinc acetate and Zinc sulphate, respectively.

**Table 5:** The effect of different zinc treatments on selected key antioxidant enzymes in *L. rohita* with S.E.

Parameters	Treatment					P-Value
	Zinc sulphate	Zinc citrate	Zinc acetate	Zinc monomethionine	Zinc gluconate	
TBARS (Liver) nMol MDA/g	2.92 ± 0.02^a^	2.45 ± 0.01^d^	2.76 ± 0.02^b^	2.52 ± 0.02^c^	2.12 ± 0.01^e^	<0.001
GPx (U/ mg Protein)	84.97 ± 0.49^c^	87.74 ± 0.51^ab^	86.61 ± 0.50^b^	87.34 ± 0.50^ab^	88.76 ± 0.51^a^	<0.001
CAT (U/ mg Protein)	25.97 ± 0.15^d^	27.94 ± 0.16^b^	26.61 ± 0.16^c^	27.59 ± 0.16^b^	28.76 ± 0.17^a^	<0.001
SOD (U/ mg Protein)	75.92 ± 0.44^c^	77.74 ± 0.45^ab^	76.55 ± 0.44b^c^	76.98 ± 0.44^bc^	78.76 ± 0.46^a^	<0.001

Superscripts differ significantly at P ≤  0.05. TBARS (Liver) nMol MDA/ g =  Thiobarbituric acid reactive substances, GPx (U/ mg Protein) =  Glutathione peroxidase, CAT (U/ mg Protein) =  Catalase, SOD (U/ mg Protein) =  Superoxide dismutase.

### Stress and immune assays

The results of the stress and immune assays showed significant (P < 0.05) differences among each of the dietary zinc treatments presented in the Table 6. HCT level was observed significantly lower in Zinc gluconate as compared to Zinc citrate, Zinc monomethionine, Zinc acetate and Zinc sulphate, respectively. On the other hand, PRT level was observed significantly higher in Zinc gluconate followed by Zinc acetate, Zinc monomethionine, and Zinc citrate as compared to Zinc sulphate, respectively. Meanwhile, GLU, and CRT levels were significantly lower in Zinc gluconate and Zinc acetate, whereas Zinc citrate and Zinc monomethionine illustrated increased levels as compared to Zinc sulphate (control).

**Table 6:** Effect of different zinc treatments on stress and immune assays in *L. rohita* with S.E.

Parameters	Treatment					P-Value
	Zinc sulphate	Zinc citrate	Zinc acetate	Zinc monomethionine	Zinc gluconate	
HCT %	36.59 ± 2.18^a^	30.49 ± 1.82^b^	28.851.42 cd	28.091.68^bc^	21.11 ± 1.26^d^	<0.01
PRT g/dl	2.75 ± 0.04^d^	3.96 ± 0.07^c^	5.13 ± 0.09^b^	4.07 ± 0.07^c^	6.13 ± 0.10^a^	<0.01
GLU g 100/ml	55.68 ± 0.0.99^a^	48.38 ± 0.86^b^	44.57 ± 0.79^c^	47.55 ± 0.85^b^	42.51 ± 0.49^c^	<0.01
CRT mg/ml	36.62 ± 0.65^a^	32.53 ± 0.58^b^	26.36 ± 0.41^c^	31.31 ± 0.55^b^	24.63 ± 0.44^c^	<0.01

Superscripts differ significantly at P ≤ 0.05. HCT, hematocrit; PRT, protein; GLU, glucose; CRT, cortisol.

### Blood serum biochemistry

Blood serum biochemical parameters of *L. rohita* fed different zinc dietary treatments are present in Table 7*.* ALT of fish fed Zinc gluconate shown significantly (P < 0.05) lower value compared to Zinc sulphate, Zinc citrate, Zinc acetate, and Zinc monomethionine, respectively. AST and ALP were shown similar trends as ALT and for the Zinc gluconate again represent lower level as compared to Zinc acetate, Zinc monomethionine, Zinc citrate, and Zinc sulphate (control), respectively.

**Table 7:** The effect of different zinc treatments on the blood serum biochemistry of *L. rohita* with S.E.

Parameters	Treatment					P-Value
	Zinc sulphate	Zinc citrate	Zinc acetate	Zinc monomethionine	Zinc gluconate	
ALT U/ml	89.34 ± 2.58^a^	63.25 ± 1.82^b^	48.45 ± 1.39^c^	38.93 ± 1.13^d^	29.56 ± 0.85^e^	<0.01
AST U/ml	190.34 ± 5.49^a^	173.23 ± 4.99^b^	110.45 ± 3.18^d^	159.54 ± 4.60^c^	84.76 ± 2.45^e^	<0.01
ALP U/ml	387.21 ± 11.17^a^	343.45 ± 9.9.91^b^	247.05 ± 7.13^d^	294.76 ± 8.51^c^	212.87 ± 6.14^e^	<0.01

Superscripts differ significantly at P ≤ 0.05. ALT, alanine amino transferase; AST, aspartate amino transferase; ALP, alkaline phosphate activity.

## Discussion

As stated previously, Zinc is a vital element required for the normal functioning of cells in living organisms and this extends to fish as recently reviewed [39] as well as by many previous workers.

These latter authors and other researchers have confirmed that different zinc treatments gave a marked improvement in the growth of *L. rohita* through better feed consumption [40–42]. The addition of different inorganic zinc sources results in the highest BWG, SGR, and FCR, as the various chemical forms of zinc may exhibit differences in bioavailability among fish species [43–46]. However, higher amounts of zinc can have a deleterious impact on fish growth due to toxicity [47–49]. Consequently, optimum dietary zinc supplementation is necessary to avoid negative impact on the fish and decrease feed cost [50, 51].

In the current study, fish proximate analysis confirmed there to be a non-significant effect in all the treatment groups. Similarly, non-significant variation recorded in the crude protein, dry matter, ash, and crude fat content [52, 53] of rohu fed different Zn supplemented diets. On the contrary, increased dry matter, improved lipid and ash contents were observed in fish fed diets with higher Zn levels [54]. This might be due to increase in anti-oxidative activities (SOD and GPx) that suppress the lipid peroxidation and consequently, lipid retention and body content would increase. The lowest and highest amount of Zn was observed in different body organs associated with the different zinc treatments. The higher tissue Zn concentration indicates a greater ability of zinc to pass across the intestinal epithelium and accumulate in body tissues, resulting in elevated zinc levels. This accumulation occurs due to the various chemical mechanisms by which each Zn species translocates zinc into target fish organs. Studies have also validated [55,56] the positive effects of zinc on growth performance, immune enzyme status, pathogen resistance, and gut microbiota in juvenile pearl gentian grouper (*Epinephelus lanceolatus*♂* × Epinephelus fuscoguttatus* ♀) fed diets low in fishmeal and primarily plant-based.

In accordance with the current study, Zn addition to diet significantly decreased TBARS content, increased SOD and GPx [58–60] activity with different dietary Zn treatments. Thus, previous findings suggest that the experimental fish may resist oxidative stress induced by minerals through antioxidant mechanisms. Zinc plays an important role as an antioxidant which affects antioxidant capacity and lipid peroxidation in many aquatic organisms [61]. Zn is the most important nutrient in the synthesis of protein, energy metabolism, immunity, and growth improvement in fish.

It should be noted that cortisol plays a role in sustaining blood glucose level by enhancing the gluconeogenesis pathways, however variation in hematocrit, cortisol and glucose levels exhibited stress response in fish [62–65]. Moreover, in relevance to the current study, HCT, CRT, GLU levels were significantly lower [66], although protein level also remained lower due to the normal (stress free) rearing environment that prevailed. This might be ascribed as under stressful conditions glucose level is raised in blood, increased adrenal cortisol and glucocorticoid secretions which perform important roles in the glucose metabolism to accomplish the instant energy requirement of fish [67]. To help meet the energy requirements of fish in stressed situations, non-essential amino acids can be synthesized *de novo* via the ALT enzyme pathway, with gluconeogenesis being promoted by the liver-specific enzyme AST [68]. In cellular nitrogen metabolism AST and ALT are essential enzymes for the amino acid oxidation and liver gluconeogenesis that might be used as biomarkers for identifying toxic conditions that initiate liver damage [69]. In the current experiment, the blood serum biochemistry parameters (ALT, AST, and ALP) remained lower in fish fed Zn gluconate. This may indicate an association with the production of a hepatic cytokine, which protects liver cells, and improved nonspecific immune functions including modulating metabolic biomarkers and enzyme antioxidant activity [70]. These could be attributed to increased Zn availability as Zn acts as a cofactor for these enzymes [71–73].

## Conclusion

In conclusion, different dietary zinc treatments produced significant impact on the growth performance, antioxidant enzyme activities, blood serum biochemistry and immune response markers of the *L. rohita*. Zn gluconate enhanced the body weight, reduced FCR, and generally improved feed utilization compared to the other sources evaluated. Furthermore, its superior absorption and retention in the body improved the efficiency of antioxidant enzyme activity, blood serum and immune related parameters. It is a superior source of zinc in terms of tissue retention and levels in the key organs evaluated compared to other zinc forms tested.

The study indicates that further research is required to optimized Zn gluconate levels needed for augmentation the growth of *L. rohita* diets with respect to phases of production towards harvest. This also has implications for human nutrition regarding trace element intake and is important for mitigating environmental concerns related to mineral waste and pollution.

## Supporting information

S1 Data Raw data. (XLSX)
